# Research on the Maturity Evaluation Model of Enterprise Safety Culture

**DOI:** 10.3390/ijerph20032664

**Published:** 2023-02-01

**Authors:** Jingjing Pei, Lu Liu, Ying Chi, Chengyang Yu

**Affiliations:** 1 School of Engineering and Technology, China University of Geosciences, Beijing 100083, China; 2 Beijing Institute of Electronic System Engineering, Beijing 100854, China; 3 Commercial Aircraft Corporation of China Flight Test Center, Shanghai 201323, China

**Keywords:** safety culture, index system, maturity model, grey fuzzy comprehensive evaluation method

## Abstract

To regulate the safety behavior of employees and improve the occupational safety level of enterprises. Based on the perspective of safety culture, this paper designed an index system based on the four dimensions of safety concept, system, behavior, and physical culture, and it explored a new quantitative assessment method of safety culture level by introducing the concept of maturity into the evaluation of safety culture using the grey fuzzy comprehensive evaluation method. Combining the characteristics of enterprise safety culture, safety culture was divided into five levels, including original level, starting level, development level, completion level, and leading level, and the maturity model of enterprise safety culture was established. Finally, taking an enterprise to be evaluated as an example, the evaluation steps and application of evaluation results were introduced. The results showed that the evaluation model of enterprise safety culture maturity constructed in this paper provides systematic measurement indexes and scientific evaluation methods for evaluating the safety culture maturity of enterprises.

## 1. Introduction

In recent years, studies on the causes of accidents in various industries and fields have consistently shown that human errors or human factors play a decisive role in accidental property losses and casualties [1,2,3,4,5,6]. Therefore, the key to strengthening accident prevention is to reduce human errors at the source, and some studies have shown that safety culture has a positive impact on the safety behavior performance of enterprise employees. Safety culture originates from the nuclear safety industry. The term “safety culture” was formally proposed after the Chernobyl nuclear power plant accident in 1986 [7]. Subsequently, in the 75-INSAG-4 report, INSAG defined safety culture for the first time as follows: “safety culture is the synthesis of various qualities and attitudes existing in units and individuals” [8]. At present, safety culture has been expanding from the nuclear industry to enterprises, and the awareness of the role of safety culture in the development of enterprise safety has gradually increased. The level of enterprise safety culture is closely related to the safety status of the enterprise [9]. Improving corporate safety culture is conducive to standardizing and guiding employees’ behaviors, reducing the occurrence of unsafe behaviors [10,11], and ultimately affects the safety performance level of enterprises [12,13].

Maturity originated in the software industry and is mainly used to describe the framework for adding or acquiring certain expectations (such as capabilities) [14]. Maturity models involve defining maturity stages or levels that assess the completeness of the analyzed objects. In November 1986, a research team at Carnegie Mellon University’s Software Engineering Institute (CMU-SEI) collected data and conducted a maturity framework study after signing a contract with the US Department of Defense to help organizations improve software processes [15]. In 1987, SEI first proposed a CMM (Capability Maturity Model of software), which was primarily used to determine the maturity level of an enterprise software process and indicate how to improve the maturity level [16,17]. When helping a software enterprise manage and improve the software engineering process, there are five levels: initial, repeatable, defined, managed, and optimized. The logic among these five levels is clear and progressive, so many industries use this idea to build maturity models for their own industries. Based on the CMM, Kerzner of the United States proposed a five-level organizational project management maturity model (K-PMMM, Kerzner-Project Management Maturity Model), in which different levels represent different levels of project management maturity, and adopted a questionnaire, scoring, and other methods to evaluate the model [18].

Scholars apply the maturity model to the field of safety and have put forward the safety culture maturity model. The essence of the safety culture maturity model should be a systematic approach to improve the positive influence of safety culture. Fleming proposed the safety culture maturity model for the first time, dividing maturity into five levels, namely: emerging stage, management stage, participation stage, cooperation stage, and sustainable development stage [19]. The classification of maturity level by scholars provides a basis for the classification of maturity level in this paper.

Studies have shown a steady increase in the use of maturity models to assess safety culture, particularly in areas such as construction, the oil and gas industry, and healthcare [20]. Filho et al. applied the maturity model to 23 petrochemical companies in Brazil to evaluate the level of safety culture of these companies [21]. In China, Zheng et al. established the safety culture maturity model by combining it with K-PMMM, applying the model to construction enterprises to quantify safety culture [22]. In summary, the current research on safety culture is more from a qualitative point of view, including the exploration of the safety culture system and framework of a certain industry or enterprise [23,24], and there is relatively little research on the quantitative assessment of safety culture. Additionally, the assessment of enterprise safety culture is generally performed with strong subjectivity, lacking the connotation analysis and objective quantitative assessment methods of enterprise safety culture. Compared with existing studies, the main contribution of this paper was to identify and define the connotation of safety culture and introduce the concept of maturity model into the evaluation of safety culture. The study of safety culture maturity can help enterprises better grasp the development direction of safety culture and formulate safety culture construction planning [25], which can play a positive and effective role in improving the safety management level and competitiveness of enterprises [26]. Moreover, the maturity model was combined with the grey fuzzy comprehensive evaluation method to explore a new objective quantitative evaluation method for safety culture level.

Therefore, in order to quantitatively evaluate the maturity of safety culture and further improve the level of enterprise safety management system and occupational safety, this paper utilized the grey fuzzy comprehensive evaluation method to construct an evaluation model of enterprise safety culture maturity. The model was used to perform a quantitative analysis of an evaluated enterprise, obtain the maturity level of its safety culture, and provide suggestions for safety culture construction.

## 2. Method

### 2.1. Expert Scoring Method

The expert scoring method was used to determine the index weight. The expert scoring method refers to the method of quantitative analysis of a certain research object after multiple rounds of consultation, feedback, and adjustment by anonymously soliciting the opinions of relevant experts, statistically processing, analyzing, and summarizing their opinions, objectively combining most experts’ experience and subjective judgments, and making reasonable estimates of a large number of factors that are difficult to quantitatively analyze by technical methods [27]. After each expert understood the enterprise’s situation and indicator system, we invited the expert to compare and score each indicator level by level, group by group, and pair by pair, thus obtaining the relative weight value of each indicator by multiple experts through calculation. The initial weight was calculated according to the relative weight value, and then the initial weight was corrected by influencing the weight to obtain the final index weight.

(1) The selected experts should be highly authoritative and representative. The experts should be professors in the field of security, enterprise security managers, and government security supervisors. They should have a high professional level, familiarity with development trends in the safety field, and have been engaged in the safety field for more than 5 years.
(2) Experts should have a good understanding of the enterprise situation and indicator system. Before grading, the grading expert should understand the meaning of each indicator and the grading criteria.
(3) The number of experts should be appropriate, with a minimum of 10.

### 2.2. Grey Fuzzy Comprehensive Evaluation Method

The grey fuzzy comprehensive evaluation method is used to comprehensively evaluate the maturity model. The grey fuzzy comprehensive evaluation method [28,29] is a combination of the fuzzy comprehensive evaluation method [30] and grey comprehensive evaluation method [31,32]. It is a method used to evaluate objects with fuzzy factors when the known information is insufficient. It can deal with fuzzy and incomplete information in the evaluation and proper sorting of schemes, achieve the evaluation and grading of schemes, and analyze and quantify scheme sorting more effectively.

The grey fuzzy comprehensive evaluation method is mainly based on the following model:

B = P × R(1)

where B is the safety culture maturity level after considering all indicators; P is the weight vector; and R is the comprehensive evaluation matrix.

The weight allocation vector P of the index system can be determined by expert scoring. To determine the comprehensive evaluation matrix, the initial matrix E should be constructed first. Then, dimensionless processing of E is performed to obtain ΔC. Finally, the comprehensive evaluation matrix R is obtained after calculating the grey relational degree.

## 3. Establishment of Evaluation Index System

### 3.1. Index System Construction

The definition of enterprise safety culture should be based on the “big safety concept” and “big culture concept”. The “big safety concept” is the concept of reasonably integrating available resources and comprehensively and systematically preventing and controlling the harm caused to people, family, and production order. The “big culture concept” is based on the height of modern culture and integrates culture into the investigation and research of human science and technology, economy, politics, society, etc. From the point of view of safety, safety culture should include enterprise, public, and family. From the point of view of culture, some scholars believe that safety culture is the sum of norms, beliefs, roles, attitudes, and behaviors within an organization and that its core concept is people-oriented and consists of social and technical dimensions [33]. Therefore, safety culture should include not only ideological (spirit, concept, etc.) but also practical and material (behavior, environment, physical state, etc.) factors.

**Figure 1:**
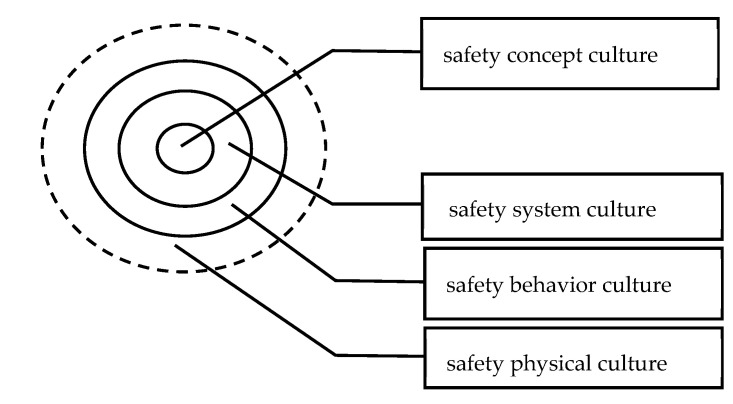
Hierarchy of Safety Culture—“Concentric Circle Theory”.

According to this definition, the maturity model of safety culture can better integrate the hardware environment and software conditions of safety culture, with more scientific levels and comprehensive indicators. At the same time, the “concentric circles” of Chinese chemistry is applied in academia, that is, culture contains three concentric circles: the outer layer is the material culture; the middle layer is the behavior culture; and the inner layer is the spiritual culture. Some scholars extend the concentric circles theory promoted from culturology to the study of safety culture, in that safety culture consists of four concentric circles, including safety physical culture, safety behavior culture, safety system culture, and safety concept culture, forming a hierarchical structure of safety culture from the outside to the inside in turn, as shown in Figure 1 [14,34]. Safety concept culture mainly refers to the safety consciousness, safety concept, and safety value standards accepted by decision makers and the public. Safety behavior culture refers to safety behavior codes, behavior patterns, and so on. Safety system culture refers to various safety rules and regulations, operating procedures, etc. Safety physical culture refers to the environmental conditions in the business activities of enterprises, etc. [30].

To comprehensively, objectively, and accurately evaluate the maturity of enterprise safety culture, the evaluation result should reflect the actual level of enterprise safety culture and understand the development stage of the enterprise’s safety culture. The selection of safety culture maturity indicators followed the indicator system, comprehensiveness, hierarchy, and other principles. According to the definition of safety culture and based on the research results of the safety culture evaluation index system of domestic scholars and research institutions [19,35,36], the index system of the initial safety culture maturity model was constructed in combination with the safety culture hierarchy. After the initial index system was built, 10 researchers in the field of safety (professor, associate professor, etc.) are invited to discuss the index system. After a one-hour meeting discussion, the final indicator system was determined, as shown in Table 1. 

### 3.2. Determination of Index Weight

In this paper, the expert scoring method was adopted to determine the initial weight and influence weight of the index, and the initial weight was modified through the influence weight to obtain the final index weight [34,37].

#### 3.2.1. Initial Weight

Considering the existence of multilevel indicators, after each expert understood the enterprise situation and indicator system, experts (at least 10) were invited to compare and grade the indicators at all levels, one by one and two by two. The relative weight values of each indicator assigned by multiple experts were obtained, given by $M_{\alpha ij}$, and then the relative weight values of each indicator assigned by multiple experts were averaged to obtain the average relative weight value of each indicator, given by $\bar{\;M_{\alpha ij}}$. Where α refers to the first level index of α, with values of A, B, C and D; i refers to the second level index of i, with values of 1, 2,…,n; and j refers to the third level index j, with values of 1, 2, …, *m*. Notably, the relative weight value of the second level indicator here only considered the relative weight value under the corresponding first level indicator dimension. Similarly, the relative weight value of the third level indicator only considered the relative weight value under the corresponding second level indicator dimension. The score was between 1 and 5, and the specific meaning is shown in Table 2.

The arithmetic mean was then used to represent the collective opinions of the experts. The calculation is as shown in Formula (2):

(2) $${M_{j}}^{\prime }=\overset{n}{\underset{i=1}{\sum }}\left({M_{ji}}^{\prime }\right)/n$$

where *n* is the number of experts (at least 10 or more according to the actual situation); *M_ji_*′ is the scoring of the (relative) weight of the *j*-th index by the *i*-th expert; and *M_j_*′ is the average (relative) weight of the *j*-th index.

The average (relative) weight of each index was then normalized to determine its (relative) weight value. The calculation formula is as set forth in Formula (3):

(3) $${M_{j}}^{\prime \prime }={M_{j}}^{\prime }/\overset{m}{\underset{j=1}{\sum }}\left({M_{j}}^{\prime }\right)$$

where *m* is the total number of indicators of this layer (Group); *Mj*′ is the average (relative) weight of the *j*-th index; and *Mj*″ is the (relative) weight of the *j*-th index.

#### 3.2.2. Influence Weight

The initial weight only reflected the importance of each group’s indicators after the comparison but did not reflect the correlation between indicators. Some of these indexes were causal, while others were interrelated, one-to-one, or one-to-many. Therefore, the direct application of the initial weight will lead to some errors in the evaluation results. On this basis, when the final weight was determined, the influence weight was introduced to modify the initial weight.

(1) Impact matrix

By analyzing the relationship between indicators and the closeness of the relationship between indicators, the influence matrix was determined, as shown in Formula (4):

(4) $$N=[\begin{array}{ccccc} b_{11} & \cdots & b_{1y} & \cdots & b_{1n}\\ \vdots & \vdots & \vdots & \vdots & \vdots \\ b_{x1} & \cdots & b_{xy} & \cdots & b_{xn}\\ \vdots & \vdots & \vdots & \vdots & \vdots \\ b_{n1} & \cdots & b_{ny} & \cdots & b_{nn} \end{array}]$$

where *N* is the influence matrix; *n* is the total number of indicators in the evaluation system; and *b_xy_* is the influence degree of *x* on *y*.

**Figure 2:**
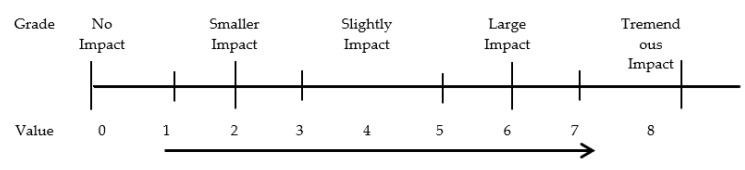
Influence Matrix Value Chart.

The *N* matrix reflects the degree of the relationship between indicators and has clear directivity. The matrix can be evaluated by experts, and the final value of each item in the matrix can be obtained by calculating the arithmetic mean value of each expert’s score. The value of *b_xy_* in the matrix was divided into 9 levels, and the corresponding situation between the value of *b_xy_* and the level is shown in Figure 2.

(2) Influence weight

After the influence matrix was determined, the influence weight of the index was determined according to Formula (5):

(5) $$M_{x2}=\frac{\sum_{x=1}^{n}\left(b_{xy}M_{y1}\right)}{\sum_{x=1}^{n}\sum_{y=1}^{n}\left(b_{xy}M_{y1}\right)}\;\;\;\;\;\;\;x\;=\;1,2,\ldots ,\;a;\;y\;=\;1,2,\ldots ,\;a$$

where *n* is the total number of indicators in the evaluation system; *b_xy_* is the influence degree of the *x*-th index on the *y*-th index; *M_y_*_1_ is the initial weight of the *y*-th index; and *M_x_*_2_ is the revised weight after considering the degree of correlation between the indicators.

#### 3.2.3. Correction Function

We modified the initial weight of the indicator with the influence weight of the indicator, as shown in Formula (6):

(6) $$M_{i}=\alpha M_{i1}+\left(1-\alpha \right)M_{i2}\;\;\;\;\;\;\;i\;=\;1,\;2\ldots ,\;n$$

where *n* is the total number of indicators in the evaluation system; *α* is the scale factor reflecting the initial weight and the influence weight, that is, the correction coefficient; *M_i_*_1_ is the initial weight of the *i*-th index; and *M_i_*_2_ is the influence weight of the *i*-th index;

It should be noted that when 0 < *α* ≤ 1, the initial weight must be considered, but when *α* = 1, the influence weight was not considered. In practical calculation, *α* = 0.5 is often taken.

Through the modification of the initial weight, the accuracy of the index weight was increased [38]. This method of weighting has both mathematical thinking and expert experience, and it is simple, easy to operate, and strong.

## 4. Maturity Level Division of Evaluation Model

**Figure 3:**
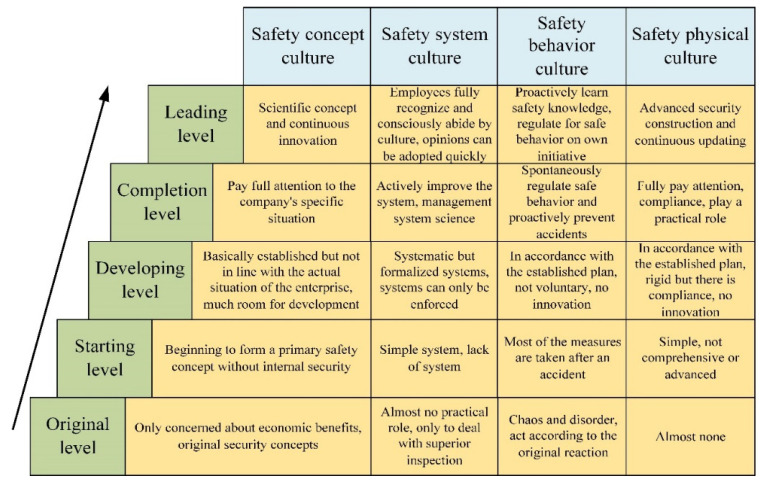
Maturity Level of Safety Culture.

Maturity refers to the relative value of the research object and its perfect state. Its main connotation has two points: one is to determine the perfect state of the object (based on the current understanding, the relative perfect state), while the other is to determine the current state of the object and the gap between it and the perfect state. The essence of the maturity model of safety culture should be a systematic method to improve the positive influence of safety culture. The current stage of safety culture can be determined by the maturity level of the safety culture of the enterprise, which further reflects the enterprise’s current safety culture to improve the occupational safety state of the incentive effect. Based on the CMM maturity model [15,20], one of the maturity research system methods is to build the maturity level of safety culture, as shown in Figure 3.

(1) Original level

The construction of enterprise safety culture is in the primitive stage. Enterprises do not have long-term development plans, the risk of accidents is very high, and enterprises are often easily eliminated by industry competition. Enterprises care solely about economic benefits, with no concept of safety; safety systems are only used for superior inspection, and there is safety behavior disorder. Attention is not paid to the construction of the safety state. At this stage, the enterprise takes a natural attitude towards the occurrence of accidents and puts all the blame on the employees involved.

(2) Starting level

Due to frequent accidents or mandatory restrictions of laws and regulations, enterprises begin to invest some resources to deal with safety problems. Enterprises begin to form a primary concept of safety, carrying out safety culture construction, but lack internal safety motivation and do not understand the importance of safety culture construction. Enterprises are under external pressure to establish a simple safety system but lack systemic systems. Most of the time, some safety behaviors and safety state construction are undertaken only after an accident occurs.

(3) Developing level

The enterprise has certain safety culture construction as well as significant development space. Enterprises at this stage can already cope with general inspection, but in-depth investigation will not reveal obvious safety culture defects before an accident occurs. The enterprise has established safety development goals, safety values, and other safety concepts, but these may not be suitable for the enterprise itself. The enterprise’s safety system and safety management system are rigid and formalized. The management and employees are forced to comply with the safety system without understanding its inherent meaning. The safety behavior management and safety material condition construction of the enterprise are carried out according to the established plan without innovation. Some enterprises may stop at this stage, but other enterprises can further develop on this basis and obtain a better virtuous circle of economy and safety.

(4) Completion level

Enterprises attach importance to the construction of safety culture and have reached the basic state of completion. Enterprises emphasize the establishment of safety commitment, safety attitude, etc. in line with enterprise development, and guide the enterprise’s internal individuals to make safety commitments. Enterprises actively improve the safety system and build a scientific safety management system, with management and staff awareness of the importance of safety, self-regulating their own safety behavior, and taking the initiative to prevent possible accidents. Enterprises attach great importance to the construction of safety state and increase safety investment.

(5) Leading level

Enterprises regard the construction of advanced safety culture with enterprise characteristics as a part of daily work, and safety issues are fully valued across and among all departments and employees without emphasis. Enterprises have a scientific concept of safety system, and continue to innovate, keeping the concept of the leading state. The safety management and system of the enterprise are recognized by employees, and reasonable suggestions of employees can be adopted in a timely manner. All employees in the enterprise take the initiative to learn new safety knowledge and consciously standardize safety behaviors. Enterprises have advanced construction of safety state; safety investment and economic benefits form a virtuous circle. 

In practical application, each stage was given a corresponding score to facilitate quantitative calculation. The experts judged the maturity stage of each indicator and gave each indicator a grade. See Table 3 for the corresponding relationship between maturity level and score.

## 5. Construction of Evaluation Standard Model

The evaluation standard model of enterprise safety culture refers to the comprehensive evaluation matrix and standard mathematical model of each enterprise standard. The result of the model corresponds to the maturity level of safety culture one by one, so it can be used as the measurement standard of the maturity level of enterprise safety culture.

### 5.1. Building of Standard Comprehensive Evaluation Matrix

#### 5.1.1. Standard Initial Matrix

The indicator set of enterprise safety culture and its four dimensions are shown in Table 4.

It was assumed that there were five standard enterprises, and the maturity stage of safety culture was at the original level, starting level, development level, completion level, and leading level, with scores of 1, 2, 3, 4, and 5, respectively. The set composed of these standard enterprises was *X* = {*x*_1_, *x*_2_, *x*_3_, *x*_4_, *x*_5_}.

Take indicator set *Y*, for example. For a specified standard enterprise *a_j_*, there are vectors *x_j_* = (*y_j_*_1_, *y_j_*_2_ …, *y_jm_*), *y_ji_*∈*y_i_*, *i* = 1, 2 …, *m*, and *j* = 1, 2 …, *n*. Where *m* is the total number of indicators, *n* is the total number of standard enterprises, and *y_ji_* represents the score of the *i*-th indicator of the *j*-th standard enterprise *a_j_*. This vector represents the value of each indicator of the enterprise. Assuming an optimal enterprise x*, each index is the optimal value. Obviously, the optimal index set of safety culture maturity level is x* = {5, 5, 5, 5}. The initial matrix *E_Y_* can be obtained from the vectors x*, x_1_, *x*_2_, *x*_3_, *x*_4_, *x*_5_, as shown in Formula (7).

(7) $$E_{Y}=[\begin{array}{cccc} 5 & 5 & 5 & 5\\ 1 & 1 & 1 & 1\\ 2 & 2 & 2 & 2\\ 3 & 3 & 3 & 3\\ 4 & 4 & 4 & 4\\ 5 & 5 & 5 & 5 \end{array}]$$

#### 5.1.2. Standard Comprehensive Evaluation Matrix

The standard initial matrix E_Y_ is dimensionless, as shown in Formula (8):

(8) $$C_{ji}=\frac{y_{ji}-y_{i}^{\min}}{y_{i}^{\max_{i}^{\min}}}\;\;\;\;\;\;\;i\;=\;1,2,\ldots ,\;m;\;j\;=\;1,2,\ldots ,\;n$$

where: *y_i_*^min^ is the minimum value of the *i*-th index *y_i_* and *y_i_*^max^ is the maximum value of the *i*-th index *y_i_*.

Dimensionless treatment of the standard initial matrix *E_Y_* yields, as shown in Formula (9):

(9) $${\Delta C}_{Y}=[\begin{array}{cccc} 1.00 & 1.00 & 1.00 & 1.00\\ 0.75 & 0.75 & 0.75 & 0.75\\ 0.50 & 0.50 & 0.50 & 0.50\\ 0.25 & 0.25 & 0.25 & 0.25\\ 0.00 & 0.00 & 0.00 & 0.00 \end{array}]$$

We calculated the grey correlation degree, as shown in Formula (10).

Taking the optimal index set C^*^ = {C^*^_1_, C^*^_2_…, C^*^_m_)} as the reference data column, the index scores *C_j_*(*j* = 1, 2…, *n*), *C_j_* = {*C_j_*_1_, *C_j_*_2_…, *C_jm_*} of each standard enterprise as the comparison data column, and using *η_j_*(*i*) to represent the correlation degree between the score *y_ji_* of the *i*-th index of the *j*-th standard enterprise *a_j_* and the *i*-th optimal index (from the enterprise x *) y_i_^*^, *η_j_*(*i*) is also called the correlation coefficient, or membership degree:

(10) $$\eta_{j}\left(i\right)=\frac{\underset{j}{min}\underset{i}{min}|C_{i}^{*}-C_{ji}|+\rho \underset{j}{max}\underset{i}{max}|C_{i}^{*}-C_{ji}|}{\left|C_{i}^{*}-C_{ji}|+\rho \underset{j}{max}\underset{i}{max}|C_{i}^{*}-C_{ji}\right|}$$

where: *ρ*∈[0,1], generally *ρ* = 0.5, is the resolution coefficient.

We constructed the standard comprehensive evaluation matrix *R_Y_*, as shown in Formula (10).

The vector of row *i* of *R_Y_* is *R_Yi_* = {*η*_1_(*i*), *η*_2_(*i*), *η*_3_(*i*), *η*_4_(*i*)}; the R is used, as shown in Formula (11):

(11) $$R_{Y}=[\begin{array}{ccccc} 0.33 & 0.40 & 0.50 & 0.67 & 1.00\\ 0.33 & 0.40 & 0.50 & 0.67 & 1.00\\ 0.33 & 0.40 & 0.50 & 0.67 & 1.00\\ 0.33 & 0.40 & 0.50 & 0.67 & 1.00 \end{array}]$$

### 5.2. Building of Standard Mathematical Model

We built a mathematical model of the maturity of enterprise safety culture, as shown in Formula (1), where *B* is the safety culture maturity level of standard enterprise *a_j_* after considering all indicators, *b_j_*∈[0,1], *j* = 1,2, …, *n*; *P* is the weight vector, and the safety culture maturity index system, *P* = (0.30, 0.25, 0.23, 0.22); and *R* is the standard comprehensive evaluation matrix.

The standard model of enterprise safety culture maturity can be calculated as *B* = (0.33, 0.40, 0.50, 0.67, 1.00).

Due to the indicator score setting, it can be obtained: *B_YA_* = *B_YB_* = *B_YC_* = *B_YD_* = *B_Y_* = *B*.

### 5.3. Level Correspondence of Safety Culture Maturity

Through the standard mathematical model, the results of the mathematical model obtained by the grey fuzzy comprehensive evaluation method corresponded to the maturity level of safety culture, and a relationship comparison table was obtained, as shown in Table 5.

According to Table 5, the calculated results of the mathematical model were between 0.330 and 1.000 when evaluating enterprises. The larger the value, the higher the maturity level of enterprise safety culture, and vice versa. It should be noted that the curved brackets represent the open interval and do not include the boundary value of the interval. The square brackets denote the closed interval, including the boundary value of the interval.

According to the results of the mathematical model of each maturity stage, we can obtain the current level of safety culture maturity and the target level of the next stage. In addition, the safety culture levels of the enterprise’s concept, system, behavior, and physical state can be analyzed according to the first level indicators.

## 6. Case Study

### 6.1. Safety Culture Maturity Evaluation

#### 6.1.1. Determination of Index Weight Vector

According to Section 3.2, the weight vector of the safety culture maturity index system was calculated as follows:

Maturity index system of safety culture: *P* = (0.30, 0.25, 0.23, 0.22).

Maturity index system of safety concept culture *A*: *PA* = (0.145, 0.143, 0.142, 0.145, 0.141, 0.142, 0.142).

Maturity index system of safety system culture *B*: *PB*= (0.111, 0.111, 0.113, 0.110, 0.110, 0.115, 0.111, 0.109, 0.110).

Maturity index system of safety behavior culture *C*: *PC* = (0.090, 0.091, 0.091, 0.092, 0.090, 0.091, 0.091,0.091, 0.090, 0.090, 0.093).

Maturity index system of safety physical culture *D*: *PD* = (0.083, 0.084, 0.084, 0.082, 0.083, 0.082, 0.084, 0.083, 0.083, 0.083, 0.085, 0.084).

According to the maturity model of safety culture, after on-site investigation, the specific scores of each evaluation index of the enterprise to be evaluated are shown in Table 6.

#### 6.1.2. Building of Comprehensive Evaluation Matrix

The standard enterprise is introduced as the calculation reference.

For standard enterprise 1, there is vector *x*_1_ = (1, 1, 1, 1) of safety culture maturity level.

For standard enterprise 5, there is vector *x*_5_ = (5, 5, 5, 5) of safety culture maturity level.

For the enterprise to be evaluated, there is vector *x_Y_* = (2.575,3.328,2.818,3.085) at the level of safety culture maturity.

From the vectors x*, *x*_1_, *x_Y_*, and *x*_5_, the initial matrix *E_Y_′* can be obtained, as shown in Formula (12).

(12) $${E_{Y}}^{\prime }=[\begin{array}{cccc} 5.000 & 5.000 & 5.000 & 5.000\\ 1.000 & 1.000 & 1.000 & 1.000\\ 2.575 & 3.328 & 2.818 & 3.085\\ 5.000 & 5.000 & 5.000 & 5.000 \end{array}]$$

The grey correlation degree was calculated and the comprehensive evaluation matrix *R_Y_′* was obtained, which is shown in Formula (13).

(13) $${R_{Y}}^{\prime }=[\begin{array}{ccc} 0.330 & 0.452 & 1.000\\ 0.330 & 0.545 & 1.000\\ 0.330 & 0.478 & 1.000\\ 0.330 & 0.511 & 1.000 \end{array}]$$

According to Formula (11), the mathematical model of safety culture maturity of the enterprise is: *B_Y_***′** = *P* · *R_Y_*′ = (0.330, 0.494, 1.000). Where 0.330 and 1.000 are the calculated reference values and 0.494 reflects the safety culture maturity level of this enterprise. According to the safety culture maturity level comparison table (Table 5), it can be concluded that the safety culture maturity level of this enterprise is currently at the starting level.

Similarly, the safety culture maturity model of the enterprise at the level of four first level indicators can be calculated:

The mathematical model of safety concept culture maturity is *B_YA_′* = (0.330, 0.458, 1.000).

The mathematical model of safety system culture maturity is *B_YB_′* = (0.330, 0.564, 1.000).

The mathematical model of safety behavior culture maturity is *B_YC_′* = (0.330, 0.488, 1.000).

The mathematical model of safety physical culture maturity is *B_YD_***′** = (0.330, 0.526, 1.000).

#### 6.1.3. Results of Analysis of Safety Culture Maturity Level

In Section 5.2, the standard model of safety culture maturity was calculated as *B* = (0.33, 0.40, 0.50, 0.67, 1.00).

According to the comparison table of safety culture levels in Table 4, the maturity level of the enterprise’s safety culture and the maturity level of each dimension were obtained, as shown in Table 7.

### 6.2. Evaluation Results and Discussion

In summary, the safety culture maturity level of the enterprise was at the starting level, but it was very close to meeting the requirements of the developing level. The safety system culture and safety physical culture in the enterprise have developed well and reached the developing level, but the safety concept and behavior culture maturity of the enterprise must be improved since their scores were lower than the overall safety culture maturity score of the enterprise and were still at the starting level.

The enterprise had a good attitude towards safety issues, a primary safety concept in the enterprise, and a vague sense of safety responsibility and honor for all its employees. The enterprise had carried out a certain degree of safety culture construction, but obviously, even the decision-making level did not have good safety behavior, which showed that the enterprise lacked internal safety power. The enterprise had established a corresponding safety system with a relatively good degree of implementation. The level of safety system culture construction was significantly higher than the level of the overall safety culture of the enterprise. However, there was no good safety information exchange in the enterprise due to the low level of safety behavior of employees; therefore, the establishment of the enterprise’s safety system may be under pressure from external inspection and the safety system was also enforced. In terms of safety physical culture construction, the enterprise had done a relatively good job; it had fully achieved safety of its equipment and tools, but it must further enrich its forms of safety publicity and display and improve employees’ sense of participation.

## 7. Discussion

### 7.1. Safety Culture Maturity Model

With increasing improvement of enterprise safety management standards, most enterprises have improved their management level and it becomes extremely difficult to stand out among many enterprises. In terms of safety, enterprises must understand their own advantages and disadvantages and set up their own competitive yardstick. Enterprises need to understand their own capabilities and position themselves so they need a complete set of methods to test and improve their capabilities. Safety culture maturity is a set of evaluation criteria applicable to measuring the safety culture capability of the enterprise itself and puts forward suggestions for improving the capability of the enterprise.

This study evaluated an enterprise using the constructed safety culture maturity model. The grey fuzzy comprehensive evaluation method was used to evaluate the safety culture maturity. Because the relationship between many indicators is grey, it is not clear which indicators are closely related and which are not. Therefore, the grey relational degree was used to describe the strength of the relationship between indicators. In this way, the relationship between indicators was comprehensively considered when evaluating safety culture, and more accurate results were obtained. 

The development speed of each dimension of safety culture is not the same [39,40]; thus, it is necessary to evaluate each dimension. The model constructed in this study can not only evaluate the maturity of safety culture but also evaluate the maturity of each dimension. In the case study, an enterprise was evaluated to obtain the scores and grades of safety culture and various dimensions (see Table 7). This helps enterprises to understand which aspects require improvement and pay more attention to those aspects in the construction of safety culture in the future.

### 7.2. Research Implications

The main purpose of this study was to build a safety culture maturity model in order to evaluate the level of enterprise safety culture maturity. The study of safety culture maturity plays an important role in promoting the establishment of safety management system and improving the competitiveness of enterprises. Therefore, a safety culture maturity model and its quantitative evaluation can effectively improve the safety culture of enterprises. This study has the following two significant theoretical findings:

This study provides a reference for the study of enterprise safety culture through the construction of safety culture maturity model. Different from other safety culture maturity models, this model is applicable to more types of enterprises. In the evaluation of enterprise safety culture maturity, a relatively accurate evaluation result can be obtained by providing a unified standard model, and the relationship between model output result and safety culture maturity level was established. The study promotes the safety culture management of enterprises.

The level of maturity was divided into five levels, namely: original level, starting level, development level, completion level, and leading level. In practical application, in order to facilitate quantitative calculation, corresponding points were assigned to each stage (see Table 3) to facilitate experts’ scoring. During the construction of the standard model, the scored segment corresponding to each level was calculated (see Table 4) to facilitate a more intuitive judgment of the safety culture maturity level.

This study provides insights for the practice of enterprise safety management. The evaluation of safety culture maturity is helpful for enterprises to better grasp the development direction of safety culture and make a safety culture construction plan. The evaluation results can provide rich feedback to help enterprises understand the advantages and disadvantages of their safety policy.

### 7.3. Limitations and Future Research Directions

In the construction of the safety culture maturity model, comprehensive coverage of the three-level indicators was ignored to a certain extent due to the need to focus on key indicators. Thus, the current evaluation model is not perfect, and further refinement of the indicators was not studied. In the future, quantitative assessment of the safety culture level will be committed to building a unified, systematic, and scientific indicator system.

In the design of the index weight, the calculated weight may have a small error due to the limitation of the number of experts. In the quantitative calculation of safety culture maturity of the evaluated enterprise, it is necessary to have some understanding of matrix calculations. This results in a large amount of computation and high requirement for computation, which may lead to some computational difficulties in practical application.

In future studies, simpler methods of calculation will be studied and simple evaluation tools will be developed. This will make the calculation process easier when quantifying the maturity of safety culture. The model will need to be extended to employees in the future to compare the results obtained from employees with those obtained from experts.

## 8. Conclusions

The following conclusions are drawn from this study:

(1) Based on safety culture hierarchy, the index system of the safety culture maturity evaluation model was designed from the four dimensions of safety concept, system, behavior, and physical culture. The initial and influence weights of the index were determined by the expert scoring method and the influence weight of the index was determined by the influence matrix, thereby modifying the initial weight and obtaining the final index weight.
(2) The maturity level of safety culture was constructed and the maturity level of enterprise safety culture was divided into original level, starting level, development level, completion level, and leading level.
(3) The grey fuzzy comprehensive evaluation method was used to evaluate the maturity of enterprise safety culture. An innovative method of constructing a standard evaluation model was used. The relationship between the output of the model and the maturity level of safety culture was established.
(4) Taking an enterprise to be evaluated as an example, this paper introduced the evaluation steps and application of the evaluation results, supplying a systematic measurement index and scientific evaluation method for enterprise safety culture evaluation.

This project provides a new idea and method for quantifying the maturity of safety culture. The evaluation of enterprise safety culture maturity is helpful for enterprises to better grasp the direction of safety culture and create a safety culture construction plan.

## Figures and Tables

**Table 1:** Index System of Safety Culture Maturity Model.

Primary Indicator	Secondary Indicator	Tertiary Indicator	Index Definition
Safety concept culture A	Safety commitment A_10_	Safety development goals and prospects A_11_	The enterprise has clear safety development goals, specific guidelines, and implements them in daily work arrangements. The current safety level of the enterprise matches its production scale and has the prospect of sustainable development.
		Safety mission and tasks A_12_	The enterprise clarifies the safety mission, tasks, and safety responsibilities that should be assumed in the industry and society. All personnel must clearly define their own safety missions and can accomplish safety tasks spontaneously.
		Full safety commitment A_13_	The company’s safety commitment is complete and accurate in content, emotionally unique and universal, and disseminated in a timely and effective manner. It is appealing. All personnel of the enterprise know and agree with the company’s safety commitment, psychologically recognize the importance of safety, and consciously abide by safety regulations.
	Safety attitude A_20_	Safety values A_21_	The enterprise can correctly handle the relationship between safety and production efficiency, has a correct understanding of the meaning of safety, and the decision-making layer has a consistent understanding of safety. The company has implemented safe production processes on its own initiative, rather than due to outside pressure.
		Safety responsibility A_22_	All employees of the company believe that safety benefits themselves, the company, their families, and society and voluntarily assume their responsibilities in the safe development of the enterprise.
	Safety concept A_30_	Safety importance A_31_	The enterprise values and maximizes safety. Safety is regarded as the first factor when allocating time, personnel, equipment, and funds. All personnel in the enterprise give priority to safety-related work.
		Safety management concept A_32_	The company has scientific and advanced safety management concepts that are in line with its actual production conditions and has slogans, systems, and documents that match these concepts. All personnel deeply understand the concept of safety management and implement it in actual production.
Safety system culture B_00_	Safety management B_10_	Management agency settings B_11_	The enterprise creates a separate safety management organization and is equipped with human and material resources in accordance with the regulations to ensure that it independently performs its duties of safety management and is responsible for full-time personnel. The decision-making level of the enterprise has a person responsible for safety management, clarifying the powers and responsibilities of the safety management organization and conforming to the actual production safety of the enterprise in terms of institutional setup and personnel arrangement.
		Division of management powers and responsibilities B_12_	The enterprise has reasonable management rights and responsibilities, and there are clear divisions of powers and responsibilities within the safety management organization.
		Safety information exchange B_13_	The enterprise establishes an internal safety information base, including safety management, safety knowledge, accident statistics, etc., and updates and improves it in a timely manner. It has specialized agencies responsible for communicating and disseminating safety information and has a stable platform and carrier for safe information management and dissemination. The company can achieve timely and active communication of safety information, and the safety instructions of superiors can be communicated efficiently. The safety suggestions and reports of employees can also be fed back to the superiors in an active, timely, and unrestricted manner. Externally, the enterprise and the government, society, industry, and family members of employees have good safety information exchange.
		Safety training and assessment B_14_	The company regularly conducts safety training for all employees, sets and updates the training content according to the actual situation of the enterprise, and arranges scientific and reasonable training hours with rich and effective training methods. The company conducts safety assessments on all employees on a regular basis and has corresponding reward and punishment measures for the assessment results. Each position has corresponding assessment requirements.
		Safety culture construction B_15_	The enterprise has a corresponding safety culture construction, regularly holds safety culture activities, and carries out safety production publicity. The enterprise establishes a sound safety culture assessment system, conducts self-evaluation of corporate safety culture on a regular basis, and regularly assesses and improves safety culture construction work based on the results of self-evaluation.
		Safety investment B_16_	The enterprise, in accordance with regulations, invests a certain proportion of personnel, funds, equipment, and other resources in safety and makes reasonable allocations. There are corresponding documents and institutions to ensure the investment and use of resources in safety.
	Safety system B_20_	Establishment of the system B_21_	The enterprise establishes scientific and reasonable safety systems within it, including a safety responsibility system, safety management system, and safety operation specifications. The establishment and improvement of the safety systems has a scientific process, and the established system reflects the advanced safety management concept and is integrated with the actual enterprise.
		Implementation of safety regulations and standards B_22_	The enterprise collects and organizes the applicable safety regulations and standards in a timely manner and strictly abides by the national and industry-related safety laws and regulations to improve its safety system and guide its safe production.
		Implementation of the system B_23_	The enterprise’s safety system has clear provisions and strict implementation. Each safety system has a mechanism for specific implementation feedback. All employees in the company can voluntarily and actively comply with the safety system.
Safety behavior culture C_00_	Decision layer behavior C_10_	Professional knowledge level C_11_	The decision-making level has the corresponding level of safety knowledge, clarifies relevant safety laws and regulations, consciously learns advanced safety concepts, performs excellently in safety assessment, and is a good example for all employees of the company.
		Establish a responsibility system C_12_	The decision-making level participates in the establishment of a safety responsibility system and clarifies the safety responsibilities of each part.
		Responsible performance C_13_	The decision-making layer earnestly fulfils its own safety responsibilities in terms of safety training, safety investment, personnel setting, system establishment, etc., and strictly abide by the principle of safety first.
		Guiding subordinates C_14_	The decision-making layer constantly increases its own level of safety knowledge, sets an example for its subordinates, and guides its subordinates to pay attention to safety from an ideological point of view and to be safe in behavior.
	Management behavior C_20_	Professional knowledge level C_21_	Management has the corresponding level of safety knowledge; clarifies relevant safety laws, regulations and policies; consciously learns advanced safety concepts; and performs excellently in safety assessment. Among them, full-time safety management personnel have professional safety capabilities and qualifications.
		Responsible performance C_22_	Management earnestly fulfills its own safety responsibilities in implementing safety systems, doing a good job in the safety management of the enterprise, and improving the enterprise’s safety performance.
		Guiding subordinates C_23_	Management certifies the safety quality of employees, goes to the site to supervise and guide the safety behavior of employees, and regularly organizes employee safety training.
	Employee behavior C_30_	Professional knowledge level C_31_	Employees have the corresponding level of safety knowledge, clarify relevant safety production rules and regulations, have the level of safety production knowledge required by the enterprise, and perform excellently in safety assessment.
		Responsible performance C_32_	Employees earnestly perform their job duties, master post-safety skills, ensure their own safe operation, have professional hazard identification and emergency response capabilities, and can supervise and protect the safety of others.
		Behavioral habit C_33_	Employees have good safety behaviors, such as strict compliance with safety systems, active communication of safety information with others, awareness of teamwork, the discovery of hidden dangers and accidents, consciously learning of various safety knowledge, earnest completion of safety training, and active participation in safety culture construction activities, etc.
		Safety performance and rewards and punishments C_34_	The enterprise establishes and continuously improves its scientific and fair safety performance and reward and punishment system. Under the premise of balancing spiritual and material rewards, it selects effective safety performance incentives and has positive effects on employees’ safety behavior improvement.
Safety physical culture D_00_	Device D_10_	Safety quality of equipment D_11_	The equipment procurement, replacement, and configuration of the enterprise are documented and require safety qualifications that are consistent with the actual production of the enterprise, and the relevant certificates are complete. Employees are familiar with the safe use of the equipment and clarify processes for handling faulty equipment.
		Equipment safety maintenance D_12_	The company regularly checks the safety and quality of the equipment and repairs or replaces equipment that has failed inspection.
		Establishment of emergency system D_13_	The enterprise establishes a timely and effective automated emergency system to ensure that equipment failures can be detected in time and ensure the safety of personnel and other equipment within the enterprise. Employees are trained in emergency response and can calmly handle accidents.
	Visualization on site D_20_	Safety alert sign setting D_21_	The enterprise posts safety warning signs in accordance with relevant laws and regulations and regularly checks to ensure that the signs are fully intact and undamaged.
		Intuitive safety information display D_22_	The company presents color and visual safety information in the locations where it is relevant, such as differences in color on a thermometer to indicate the temperature. The company visually and intuitively shows employees the safety requirements and information for each position to ensure safe operation by employees.
	Tools and protective equipment D_30_	Safety equipment D_31_	The company is equipped with tools with safety protection functions, regularly inspects and maintains the tools used by employees, and employees are proficient in the safe use of tools.
		Use of safety equipment D_32_	The company arranges comfortable and safe protective equipment for employees according to regulations and fully considers the individual needs of employees, such as different types of gloves and masks.
	Promotional display D_40_	Display mode D_41_	The enterprise uses a variety of presentation methods to promote safety knowledge, such as banners, posters, bulletin boards, and electronic displays.
		Display content D_42_	The company’s display content is rich and has educational significance, such as national laws and regulations, on-site operational safety procedures, and advanced personal deeds of corporate safety. The company regularly updates the display content, which is in line with the company’s safety culture and safe production practices.
		Place of display D_43_	The company has a suitable display location that does not affect normal production work and is noticed by employees.
	Business environment D_50_	Working environment D_51_	The company has a good working environment to ensure the health and wellbeing of employees. The work area is regularly cleaned. The equipment is neatly arranged. The employees have sufficient working space that meets the requirements of national laws and regulations.
		Rest environment D_52_	The company is equipped with comfortable and clean staff quarters, canteens, safety training areas, cultural and sports activities areas, etc. The equipment and building area of each division is in line with the actual number of employees in it, with good greening in the plant area.

**Table 2:** Initial Weight Assignment.

Implication	Most Important	Important	More Important	Slightly Important	Unimportant
points	5	4	3	2	1

**Table 3:** Maturity Stage Corresponding to Index Score.

Maturity Level	Original Level	Starting Level	Developing Level	Completion Level	Leading Level
Indicator score interval	1	2	3	4	5

**Table 4:** Corresponding Table of Each Indicator Set.

	Index Set
Safety culture	*Y* = {*y*_1_, *y*_2_, *y*_3_, *y*_4_}
Safety concept culture A	*Y_A_* = {*y_A_*_1_, *y_A_*_2_, *y_A_*_3_, *y_A_*_4_, *y_A_*_5_, y_A6_, *y_A_*_7_}
Safety system culture B	*Y_B_* = {*y_B_*_1_, *y_B_*_2_, *y_B_*_3_, *y_B_*_4_, *y_B_*_5_, *y_B_*_6_, *y_B_*_7_, *y_B_*_8_, *y_B_*_9_}
Safety behavior culture C	*Y_C_* = {*y_C_*_1_, *y_C_*_2_, *y_C_*_3_, *y_C_*_4_, *y_C_*_5_, *y_C_*_6_, *y_C_*_7_, *y_C_*_8_, *y_C_*_9_, *y_C_*_10_, *y_C_*_11_}
Safety physical culture D	*Y_D_* = {*y_D_*_1_, *y_D_*_2_, *y_D_*_3_, *y_D_*_4_, *y_D_*_5_, *y_D_*_6_, *y_D_*_7_, *y_D_*_8_, *y_D_*_9_, *y_D_*_10_, *y_D_*_11_, *y_D_*_12_}

**Table 5:** Comparison of Safety Culture Maturity Levels.

Maturity Level	Original Level	Starting Level	Developing Level	Completion Level	Leading Level
Results of the Model	[0.33,0.40)	[0.40,0.50)	[0.50,0.67)	[0.67,1.000)	1.000

**Table 6:** The Corresponding Table of Each Indicator Set of The Enterprise to be Evaluated.

	Index Set
Safety culture	*Y* = {2.575, 3.328, 2.818, 3.085}
Safety concept culture A	*Y_A_* = {3, 3, 2, 3, 2, 3, 2}
Safety system culture B	*Y_B_* = {4, 3, 2, 4, 3, 3, 4, 4, 3}
Safety behavior culture C	*Y_C_* = {3, 3, 2, 3, 4, 3, 3, 2, 2, 3, 3}
Safety physical culture D	*Y_D_* = {3, 4, 3, 3, 3, 4, 3, 2, 3, 2, 4, 3}

**Table 7:** Enterprise Safety Culture Maturity Scale to be Evaluated.

	Score	Level
Safety culture maturity	0.494	Starting level
Maturity of safety concept culture	0.458	Starting level
Maturity of safety system culture	0.564	Developing level
Maturity of safety behavior culture	0.488	Starting level
Maturity of safety physical culture	0.526	Developing level

## Data Availability

Not applicable.
